# Temporal trends and disparities in heat-related cardiovascular mortality in the United States (1999–2024): a CDC WONDER analysis

**DOI:** 10.3389/fcvm.2026.1850364

**Published:** 2026-06-24

**Authors:** Hassaan Abid, Gaaitri Lohano, Muhammad Vazaym, Muhammad Jawad, Rimsha Adnan, Muhammad Mohid Haroon

**Affiliations:** 1 Department of Internal Medicine, Indiana University School of Medicine, IN, Muncie, Indiana, United States; 2 Department of Internal Medicine, Liaquat University of Medical and Health Sciences, Jamshoro, Pakistan; 3 Department of Internal Medicine, Dow University of Health Sciences (DUHS), Karachi, Pakistan; 4 Department of Internal Medicine, Ameer-Ud-Din Medical College, Lahore, Pakistan

**Keywords:** cardiovascular mortality, CDC WONDER, epidemiology, heat exposure, joinpoint regression, mortality trends

## Abstract

Rising global temperatures and increasing frequency of extreme heat events have raised growing concerns regarding heat-related cardiovascular mortality. Heat exposure can exacerbate underlying cardiovascular conditions and precipitate acute adverse events. This study aimed to evaluate national trends in heat-related cardiovascular mortality in the United States from 1999 to 2024. We conducted a retrospective analysis using the CDC WONDER Multiple Cause-of-Death database. Deaths among adults aged ≥25 years with heat-related conditions (ICD-10 code T67) listed as contributing causes and diseases of the circulatory system (ICD-10 codes I00–I99) as underlying causes were identified. Age-adjusted mortality rates (AAMRs) per 100,000 population were calculated using the 2000 U.S. standard population. Temporal trends were analyzed using Joinpoint regression to estimate annual percent change (APC) and average annual percent change (AAPC). A total of 10,731 deaths were identified. Mortality declined significantly between 1999 and 2004, followed by variable trends, with a sharp and statistically significant increase observed from 2015 to 2024 (APC: 17.58%; *p* < 0.001). Despite a non-significant overall trend (AAPC: 1.47%; *p* = 0.79), subgroup analyses revealed important disparities. Males demonstrated a significant overall increase in mortality (AAPC: 3.64%; *p* = 0.034), while Hispanic populations exhibited the most pronounced recent increases. Regional analysis revealed the highest burden in the Western and Southern United States. Heat-related cardiovascular mortality has increased substantially in recent years, with significant demographic and geographic disparities, highlighting the growing impact of climate-related heat exposure and the need for targeted public health interventions.

## 1 Introduction

Heat-related illness and death have become increasingly important public health issues in the United States, largely due to rising global temperatures and more frequent extreme heat events (1). Elevated ambient temperatures can precipitate a range of cardiovascular complications, including myocardial infarction, arrhythmias, and stroke, particularly in individuals with underlying heart disease. In addition to classic heat-related conditions such as heat exhaustion and heat stroke, heat exposure imposes significant physiological stress. Mechanisms such as dehydration, peripheral vasodilation, reduced central blood volume, and increased cardiac workload can disrupt cardiovascular stability and contribute to acute clinical deterioration (2–4).

A growing body of epidemiological research has demonstrated a clear relationship between high temperatures and increased mortality, especially deaths related to circulatory system diseases (2, 5). Heat exposure may function not only as a direct cause of death but also as a contributing factor that accelerates mortality in vulnerable individuals with pre-existing cardiovascular conditions. Data from CDC WONDER Multiple Cause-of-Death records indicate that heat-related mortality has risen across a wide range of demographic and geographic groups in the United States (4, 5). These findings highlight the importance of considering both underlying and contributing causes of death, as analyses limited to underlying causes alone may underestimate the true burden of heat exposure on population health (6, 7).

Examining national mortality trends provides valuable insight into at-risk populations and allows for the identification of disparities across age, sex, race/ethnicity, and urban–rural classifications (4, 5). Such information is critical for informing public health responses, including the development of heat warning systems and targeted prevention strategies. This is particularly relevant given projections that climate change will continue to increase the frequency, intensity, and geographic spread of extreme heat events in the United States (7).

Although prior studies have established a link between heat exposure and cardiovascular mortality, there remains a lack of comprehensive national analyses that incorporate heat as a contributing factor alongside underlying circulatory disease. Accordingly, this study aims to evaluate trends in heat-related deaths associated with cardiovascular conditions in the United States from 1999 to 2024, with a focus on variations by age, sex, race/ethnicity, geographic region, and urban–rural status, in order to better identify high-risk populations and guide targeted public health interventions.

## 2 Materials and methods

### 2.1 Study setting and population

This retrospective analysis used data obtained from the Centers for Disease Control and Prevention's Wide-ranging Online Data for the Epidemiologic Research (CDC WONDER) database to assess the U.S. mortality trends with heat-related cardiovascular mortality from 1999 to 2024 (8). Records from the Multiple Cause-of-Death (MCD) were analysed for decedents aged ≥25 years and were subdivided into young adults (24–44 years), middle-aged adults (45–64 years), and older adults (65 + years). The Multiple Cause-of-Death Public Use record death certificates were studied to identify records in which heat related deaths were mentioned as contributing causes of death on nationwide death certificates. Whereas, diseases of the circulatory system were mentioned as underlying cause of death. Heat-related deaths were identified with the International Classification of Diseases 10th Revision Clinical Modification MCD - ICD-10 Codes: T67 and I00-I99 for diseases of the circulatory system, respectively. This approach using multiple cause-of-death data was chosen to better capture the contribution of heat exposure to cardiovascular mortality, which may be underrepresented when only underlying causes are analyzed. This study was exempt from local Institutional Review Board approval because the CDC WONDER database contains publicly available and anonymized data. All methods used in our study followed the STROBE guidelines.

### 2.2 Data abstraction

Data extracted comprised variables such as year of death, demographic factors like age, sex, and race/ethnicity; U.S. state of residence and geographical region classification; and urbanicity. The places of death were classified as medical facilities (including outpatient, emergency room, inpatient, death on arrival, or status unknown), home, hospice, and long-term care facilities. The age categories included three predefined groups: young adults (9–28), middle-aged adults (45–64), and older adults (65+). Race and ethnicity categories were restricted to Hispanics/Latinos, NH White, and NH Black or African American only due to reliability concerns. The data for race was included from 2009 to 2024. Data before 2009 was excluded due to reliability concerns. Geographic variable included the census regions designated according to the U.S. Census Bureau's regional designations (Northeast, Midwest, South, and West), which were available for the entire study period (1999–2024), while county-level urbanization classifications, which were available only for 1999–2020; data beyond 2020 were excluded due to reliability concerns. The 2013 National Center for Health Statistics Metropolitan Non-metropolitan classification Scheme was applied to assign Urban and Rural classification (29). The study was conducted in accordance with the STROBE (Strengthening the Reporting of Observational Studies in Epidemiology) guidelines (30), and reliance on anonymized, publicly accessible data, it was exempt from IRB approval.

### 2.3 Statistical analysis

Age-adjusted mortality rates (AAMRs) per 100,000 population were calculated using the 2000 U.S. standard population. Crude mortality rates (CMRs) were calculated by dividing the number of heat-related cardiovascular deaths by the corresponding U.S. population for each year. Temporal trends were analyzed using Joinpoint Regression Program (Version 5.0, National Cancer Institute). Annual percent change (APC) and average annual percent change (AAPC) were calculated with 95% confidence intervals. A two-sided *p*-value < 0.05 was considered statistically significant.

## 3 Results

### 3.1 Overall

**Figure 1:**
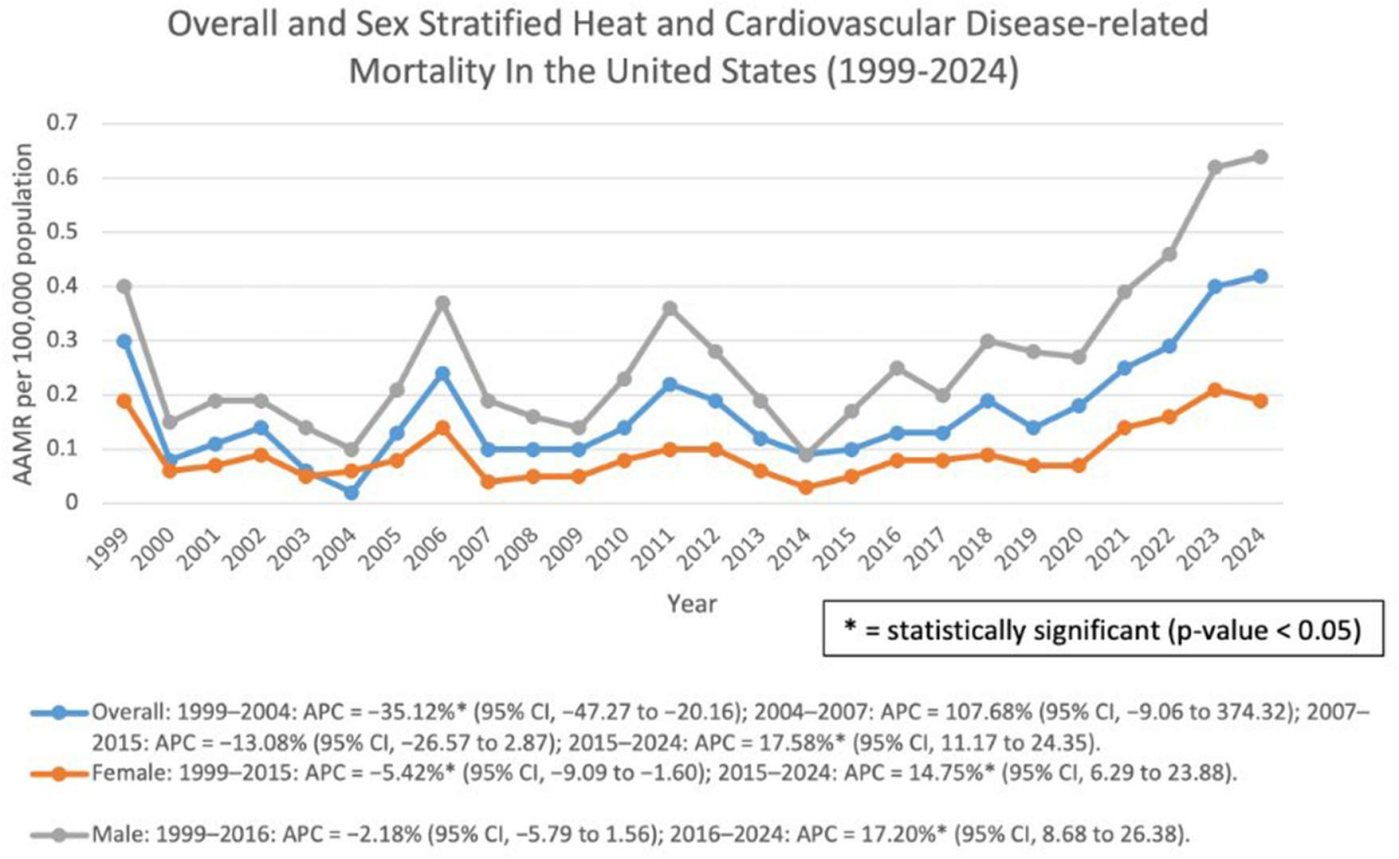
Overall and Sex-stratified AAMR per 100,000 in the United States, 1999-2024.

Between 1999 and 2024, a total of 10,731 deaths were recorded in the United States (US). During this period, age-adjusted mortality rates (AAMRs) demonstrated considerable temporal variability with multiple joinpoints. A significant decline in mortality was observed from 1999 to 2004 (APC: −35.12%; 95% CI: −47.27 to −20.16; *p* = 0.000473), followed by a non-significant increase from 2004 to 2007 (APC: 107.68%; 95% CI: −9.06 – 374.32; *p* = 0.078785). This was succeeded by a non-significant decline from 2007 to 2015 (APC: −13.08%; 95% CI: −26.57 – 2.87; *p* = 0.096421). From 2015 to 2024, a sharp and statistically significant increase in mortality was observed (APC: 17.58%; 95% CI: 11.17 – 24.35; *p* = 0.000018). Overall, the AAMR demonstrated a statistically non-significant trend over the full study period, with an average annual percentage change (AAPC) of 1.47% (95% CI: −9.29 – 13.50; *p* = 0.798790) (Figure 1).

### 3.2 Gender

Stratification of data by gender revealed distinct temporal trends in mortality. Among females, a statistically significant decline in mortality was observed from 1999 to 2015 (APC: −5.42%; 95% CI: −9.09 to −1.60; *p* = 0.007962), followed by a significant increase from 2015 to 2024 (APC: 14.75%; 95% CI: 6.29 – 23.88; *p* = 0.001209). However, the overall trend across the full study period was statistically non-significant (AAPC: 1.39%; 95% CI: −2.12 – 5.03; *p* = 0.442452).

In contrast, the male population demonstrated a non-significant decline in mortality from 1999 to 2016 (APC: −2.18%; 95% CI: −5.79 – 1.56; *p* = 0.235564), followed by a sharp and statistically significant increase from 2016 to 2024 (APC: 17.20%; 95% CI: 8.68 – 26.38; *p* = 0.000264). Overall, males exhibited a statistically significant increasing trend over the full study period (AAPC: 3.64%; 95% CI: 0.26 – 7.13; *p* = 0.034368) (Figure 1).

### 3.3 Race/ethnicity

Analysis of mortality trends was stratified by ethnicity [Hispanic or Latino vs. Non-Hispanic (NH) origin] and further categorized into racial groups: NH White and NH Black or African American.

Among Hispanic or Latino individuals, mortality trends demonstrated an initial non-significant decline from 2009 to 2019 (APC: −1.09%; 95% CI: −7.91 – 6.22; *p* = 0.739654), followed by a sharp and statistically significant increase from 2019 to 2024 (APC: 26.65%; 95% CI: 13.20 – 41.70; *p* = 0.000724). Overall, Hispanic or Latino individuals exhibited a statistically significant increasing trend over the study period (AAPC: 7.40%; 95% CI: 1.76 – 13.35; *p* = 0.009447).

Among NH Black or African American individuals, mortality trends showed a non-significant increase from 2009 to 2011 (APC: 38.33%; 95% CI: −29.45 – 171.26; *p* = 0.298712), followed by a significant decline from 2011 to 2015 (APC: −27.02%; 95% CI: −43.11 to −6.38; *p* = 0.019382). This was succeeded by a statistically significant increase from 2015 to 2024 (APC: 15.58%; 95% CI: 10.30 to 21.11; *p* = 0.000098). However, the overall trend across the full study period was not statistically significant (AAPC: 4.72%; 95% CI: −5.04 – 15.49; *p* = 0.355312).

**Figure 2:**
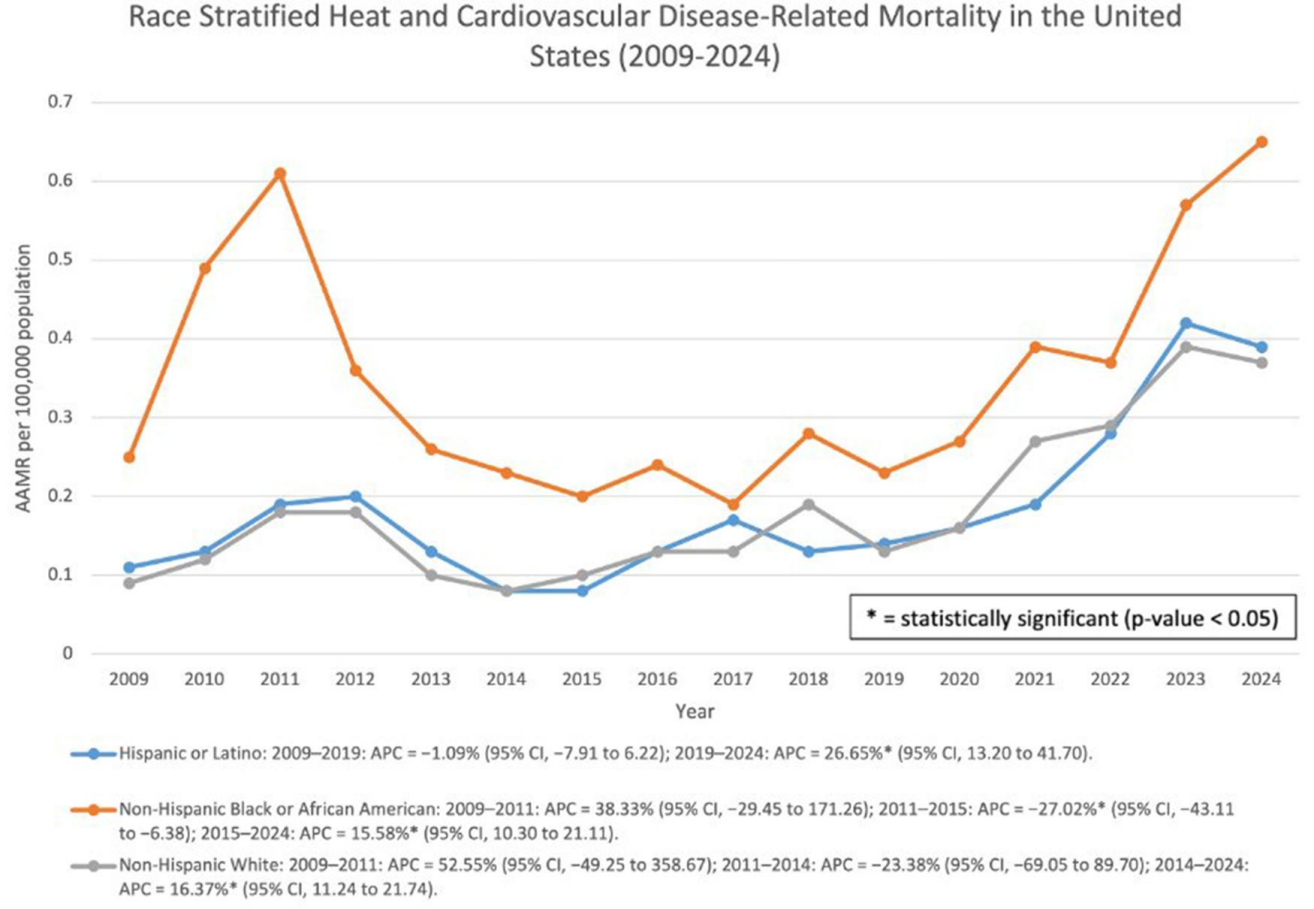
Ethnicity-stratified AAMR per 100,000 in the United States, 1999-2024.

Among NH White individuals, mortality trends demonstrated a non-significant increase from 2009 to 2011 (APC: 52.55%; 95% CI: −49.25 – 358.67; *p* = 0.402073), followed by a non-significant decline from 2011 to 2014 (APC: −23.38%; 95% CI: −69.05 – 89.70; *p* = 0.517170). From 2014 to 2024, a sharp and statistically significant increase in mortality was observed (APC: 16.37%; 95% CI: 11.24 – 21.74; *p* = 0.000055). Overall, the trend remained statistically non-significant across the study period (AAPC: 10.97%; 95% CI: −9.13 – 35.53; *p* = 0.307321) (Figure 2).

### 3.4 Age group

**Figure 3:**
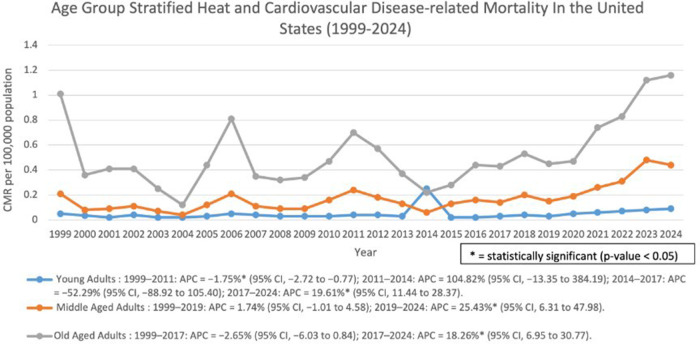
Age-stratified CMR per 100,000 in the United States, 1999-2024.

The mortality trends were divided into three age groups: young adults (25–44 years), middle age adults (45–64 years), and older adults (65–85 + years). Among older adults, a non-significant decline in mortality was observed from 1999 to 2017 (APC: −2.65%; 95% CI: −6.03 – 0.84; *p* = 0.127754), followed by a sharp and statistically significant increase from 2017 to 2024 (APC: 18.26%; 95% CI: 6.95 – 30.77; *p* = 0.002288). However, the overall trend across the study period was not statistically significant (AAPC: 2.79%; 95% CI: −0.81 – 6.53; *p* = 0.130794). In the middle age population, a non-significant increase in mortality was observed from 1999 to 2019 (APC: 1.74%; 95% CI: −1.01 – 4.58; *p* = 0.205201), followed by a statistically significant increase from 2019 to 2024 (APC: 25.43%; 95% CI: 6.31 – 47.98; *p* = 0.009584). Overall, middle age adults exhibited a statistically significant increasing trend over the full study period (AAPC: 6.09%; 95% CI: 2.19 – 10.14; *p* = 0.001954). Individuals among the younger age group demonstrated a statistically significant decline in mortality from 1999 to 2011 (APC: −1.75%; 95% CI: −2.72 to −0.77; *p* = 0.001702), followed by a non-significant increase from 2011 to 2014 (APC: 104.82%; 95% CI: −13.35 – 384.19; *p* = 0.095947). This was succeeded by a non-significant decline from 2014 to 2017 (APC: −52.29%; 95% CI: −88.92 to 105.40; *p* = 0.296936), and a sharp statistically significant increase from 2017 to 2024 (APC: 19.61%; 95% CI: 11.44 – 28.37; *p* = 0.000074). However, the overall trend across the study period was not statistically significant (AAPC: 3.96%; 95% CI: −13.84 – 25.45; *p* = 0.685232) (Figure 3).

### 3.5 Geographic regions

**Figure 4:**
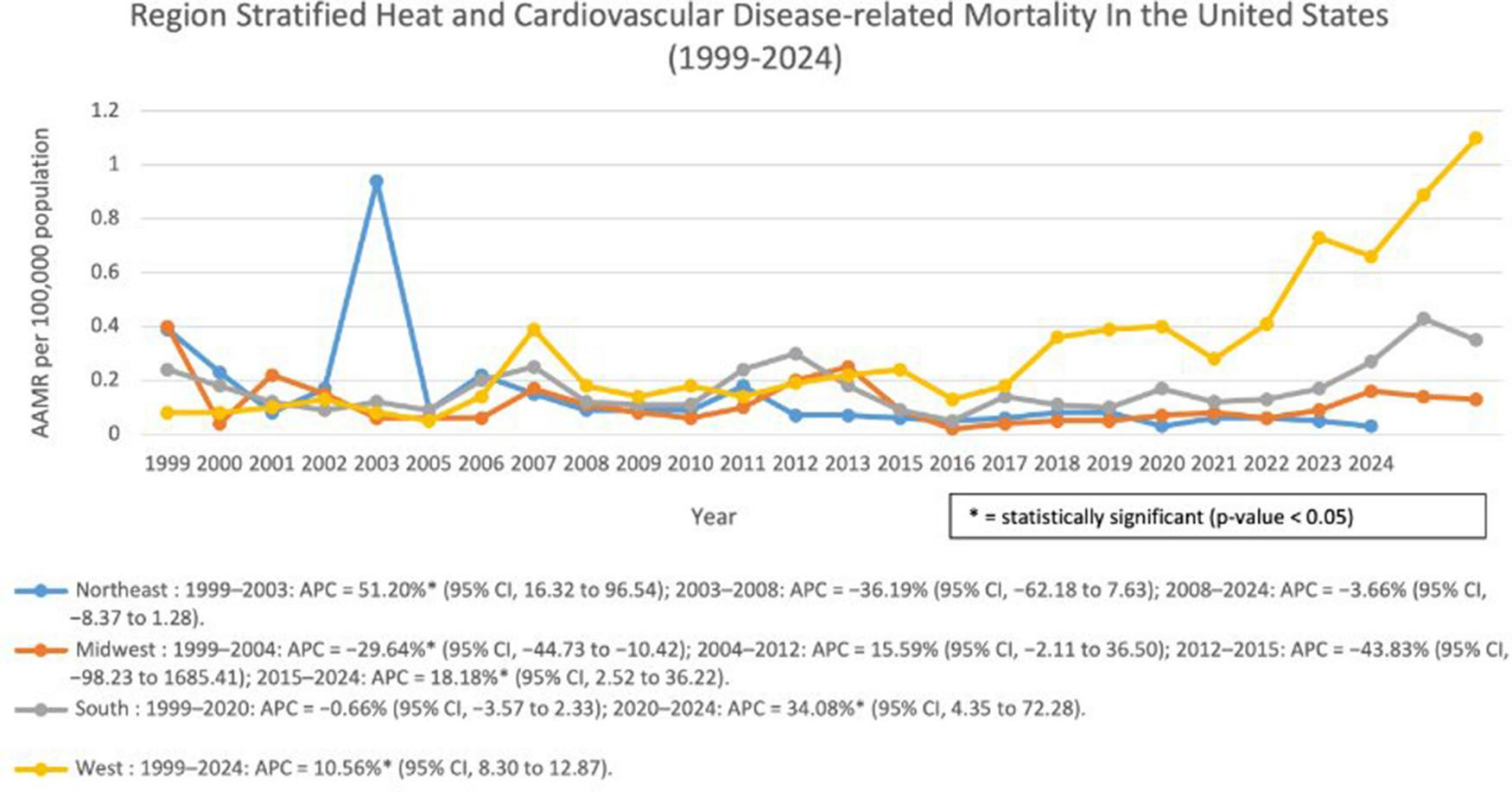
Census-stratified AAMR per 100,000 in the United States, 1999-2024.

Database considering mortality trends was segregated into four geographical areas: Northeast, Midwest, South, and Western regions. In the Northeast region, a statistically significant increase in mortality was observed from 1999 to 2003 (APC: 51.20%; 95% CI: 16.32–96.54; *p* = 0.004134), followed by a non-significant decline from 2003 to 2008 (APC: −36.19%; 95% CI: −62.18–7.63; *p* = 0.087260). From 2008 to 2024, a continued non-significant decline in mortality was observed (APC: −3.66%; 95% CI: −8.37–1.28; *p* = 0.133409). Overall, the trend across the full study period was not statistically significant (AAPC: −4.65%; 95% CI: −14.44–6.25; *p* = 0.388613). In the Midwest region, a statistically significant decline in mortality was observed from 1999 to 2004 (APC: −29.64%; 95% CI: −44.73 to −10.42; *p* = 0.007279), followed by a non-significant increase from 2004 to 2012 (APC: 15.59%; 95% CI: −2.11–36.50; *p* = 0.082992). This was succeeded by a non-significant decline from 2012 to 2015 (APC: −43.83%; 95% CI: −98.23–1685.41; *p* = 0.727175), and a statistically significant increase from 2015 to 2024 (APC: 18.18%; 95% CI: 2.52–36.22; *p* = 0.024266). However, the overall trend remained non-significant (AAPC: −3.25%; 95% CI: −34.50–42.93; *p* = 0.868188). In the South region, a non-significant change in mortality was observed from 1999 to 2020 (APC: −0.66%; 95% CI: −3.57–2.33; *p* = 0.645292), followed by a sharp and statistically significant increase from 2020 to 2024 (APC: 34.08%; 95% CI: 4.35–72.28; *p* = 0.023992). The overall trend approached but did not reach statistical significance (AAPC: 4.21%; 95% CI: −0.32–8.96; *p* = 0.069098). In the Western region, a steady and statistically significant increase in mortality was observed throughout the entire study period from 1999 to 2024 (APC: 10.56%; 95% CI: 8.30–12.87; *p* < 0.000001). This corresponded to a statistically significant increasing trend overall (AAPC: 10.56%; 95% CI: 8.30–12.87; *p* < 0.000001) (Figure 4).

### 3.6 Urbanization

**Figure 5:**
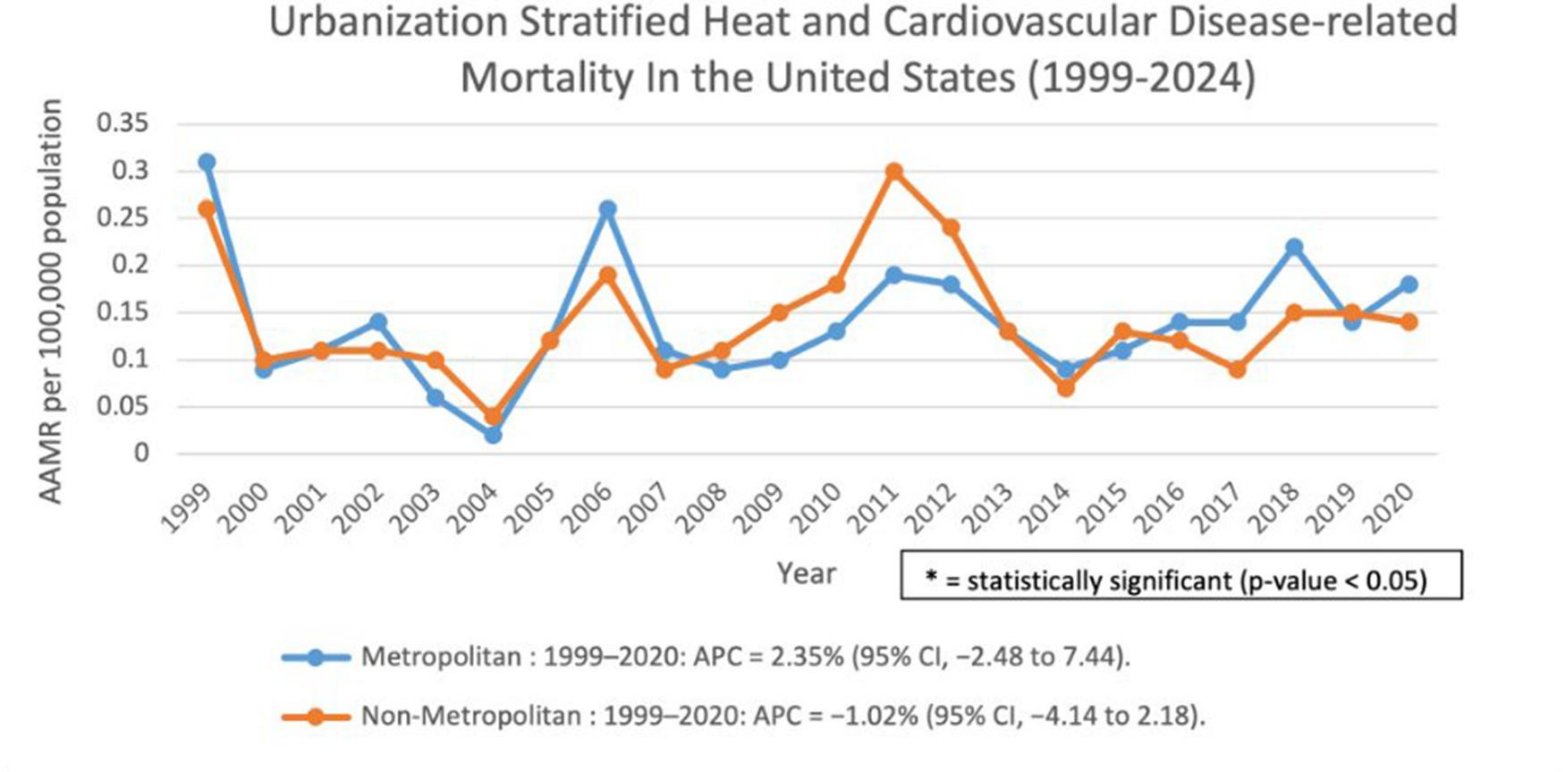
Urbanization-stratified AAMR per 100,000 in the United States, 1999-2024.

Stratification of mortality trends by urbanization revealed differences between metropolitan and non-metropolitan areas. In metropolitan areas, a non-significant increase in mortality was observed from 1999 to 2020 (APC: 2.35%; 95% CI: −2.48–7.44; *p* = 0.327839). Similarly, the overall trend across the study period remained statistically non-significant (AAPC: 2.35%; 95% CI: −2.48–7.44; *p* = 0.327839). In non-metropolitan areas, a non-significant decline in mortality was observed from 1999 to 2020 (APC: −1.02%; 95% CI: −4.14–2.18; *p* = 0.507576). The overall trend also remained statistically non-significant (AAPC: −1.02%; 95% CI: −4.14–2.18; *p* = 0.507576) (Figure 5).

### 3.7 Place of death

**Figure 6:**
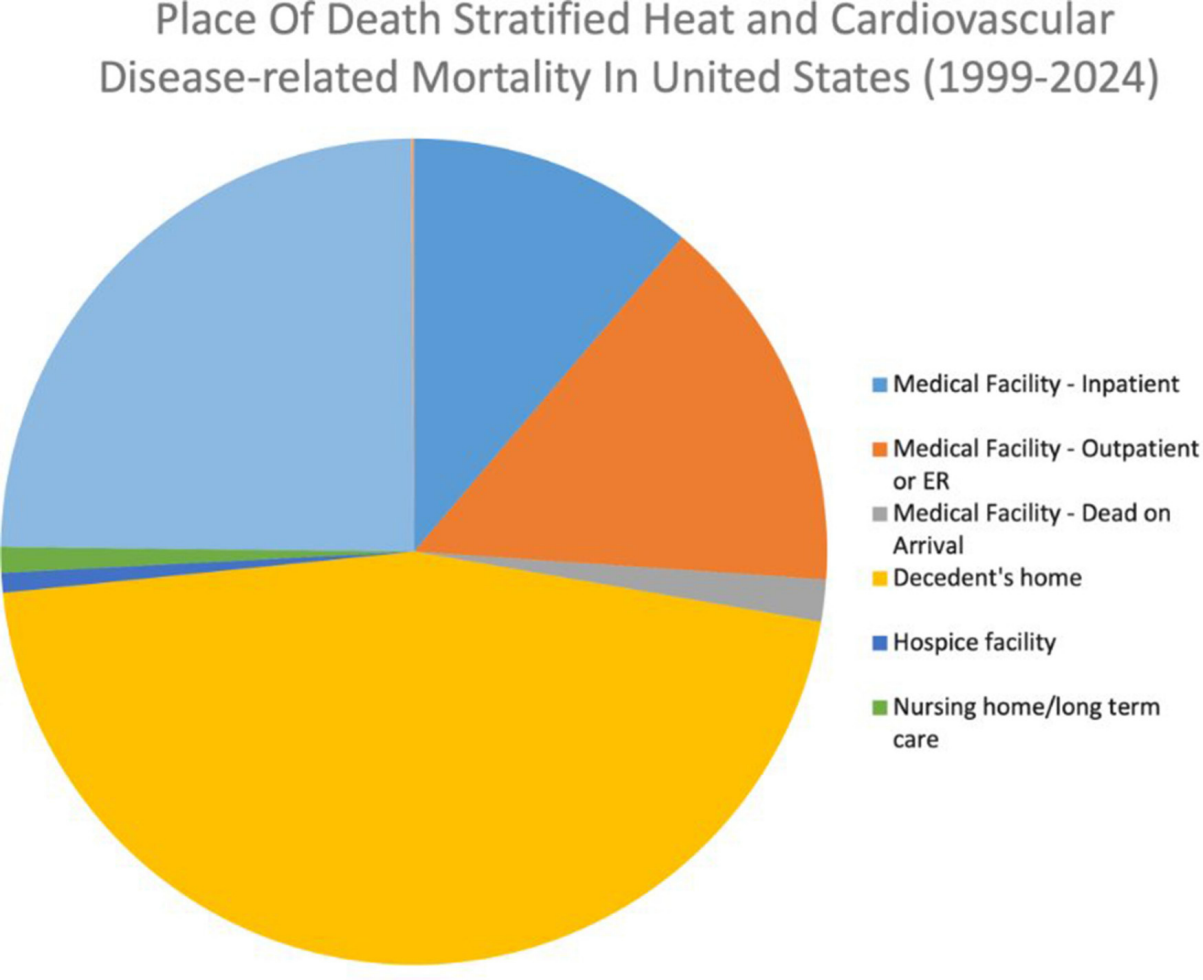
Deaths by place of death in the United States, 1999-2024.

Analysis of place of death revealed that the majority of deaths occurred at the decedent's home (4,901), followed by other locations (2,650), and medical facilities–outpatient or emergency room (1,591). Deaths occurring in medical facilities–inpatient accounted for 1,207 cases, while medical facility–dead on arrival contributed to 176 deaths. Relatively fewer deaths were observed in nursing homes or long-term care facilities (108) and hospice facilities (83), while a minimal number of cases had an unknown place of death (31) (Figure 6).

## 4 Discussion

In this nationwide analysis of heat-related cardiovascular mortality in the United States from 1999 to 2024, we observed dynamic temporal patterns characterized by an early decline followed by a marked and sustained increase beginning in 2015. Although the overall long-term trend did not reach statistical significance, the recent acceleration in mortality highlights an emerging and clinically important public health concern. By incorporating heat exposure as a contributing cause of death, this study provides a more comprehensive estimate of cardiovascular mortality burden, which may be underestimated when analyses are limited to underlying causes alone (6, 7).

Our analysis revealed substantial disparities across demographic groups. Males consistently exhibited higher mortality rates and a statistically significant overall increase, consistent with prior research (5).

This disparity is likely due to increased vulnerability related to occupational heat exposure, higher baseline cardiovascular risk, and differences in healthcare utilization (7, 32, 33) Consistent with this, Ha et al. reported that males were more likely to be hospitalized during periods of extreme heat exposure. This observation aligns with a systematic review of stroke epidemiology, which demonstrated a 33% higher incidence and a 41% higher prevalence of stroke among men compared with women, suggesting a greater underlying cardiovascular risk burden in males (20, 21). Additionally, hormones play an important role in central and peripheral thermoregulation. Estrogen enhances heat dissipation through vasodilation and a lower sweating threshold, whereas progesterone promotes heat conservation. These hormonal differences influence fluid homeostasis and thermoregulatory responses, potentially increasing male susceptibility to high temperatures due to the absence of these protective effects (22, 23).While females demonstrated lower overall mortality, the rising trend in recent years suggests increasing susceptibility, potentially driven by the growing prevalence of cardiometabolic conditions such as obesity, hypertension, and diabetes (7, 34, 35).

Age-stratified analyses confirmed that older adults remain the most vulnerable population, likely due to impaired thermoregulation, higher comorbidity burden, and medication-related susceptibility (36–38). However, the increasing mortality observed among middle-aged and younger adults suggests that heat-related cardiovascular risk is expanding beyond traditionally high-risk populations, possibly reflecting cumulative environmental exposure and evolving lifestyle patterns (39, 40).

Racial and ethnic disparities further underscore the unequal burden of heat-related cardiovascular mortality. NH Black individuals demonstrated the highest mortality rates throughout the study period, followed by Hispanic or Latino and NH White individuals. These findings are consistent with prior research (5, 24) and likely reflect structural and socioeconomic determinants, including occupational exposure, housing conditions, access to cooling resources, and disparities in healthcare access (5, 41). Black individuals have a higher burden of cardiovascular risk factors which can be exacerbated due to extreme heat conditions and lead to adverse effects (25, 26). Research has indicated that compared with NH Whites, NH Black individuals experienced a fourfold increase in the proportion of excess cardiovascular disease deaths per population for each additional day of extreme heat per month (5). Moreover, African Americans and other minority groups have lower socioeconomic status, poorer baseline health, reduced access to air conditioning, a higher likelihood of employment in outdoor occupations such as agricultural work, and residence in neighborhoods with limited vegetation and more heat retaining surfaces, known as heat islands (27, 28, 42–44). Hispanic populations demonstrated the most pronounced recent increases, particularly between 2019 and 2024 and are likely due to the occupational exposure, especially in states with extensive agricultural and construction industries where Hispanic individuals are overrepresented in outdoor work (41, 45, 46). Additionally, the COVID-19 pandemic affected healthcare-seeking behavior, as COVID-19 case rates were highest in Black and Hispanic communities, potentially contributing to increased reluctance to seek hospital care for acute conditions, which may have further exacerbated cardiovascular outcomes during this period (47, 48). A survey by the American Heart Association revealed that 41% of Hispanic Americans and 33% of Black Americans avoided seeking hospital care despite suspecting a heart attack or stroke, primarily because of concerns about COVID-19 exposure (49). These findings underscore the need for equitable heat mitigation strategies, targeted occupational protections, and improved access to preventive cardiovascular care in racially and ethnically vulnerable communities disproportionately affected by extreme heat. Geographic analyses revealed that the Western and Southern United States bear the greatest burden of mortality. These regions experience higher frequency and intensity of extreme heat events and are more susceptible to urban heat island effects, which may amplify population-level exposure and risk (50, 51). The consistent and statistically significant increase observed in the Western region is particularly notable and may reflect both climatic trends and demographic vulnerability.

Urbanization patterns demonstrated modest increases in mortality in metropolitan areas, potentially attributable to the urban heat island effect, where built environments retain and amplify heat exposure (9). Cleland et al. reported that areas with high urban heat island intensity (UHII) contributed to 35% of all heat-related cardiovascular disease burden (9). Moreover, socioeconomic inequalities increase vulnerability to heat-related illness and mortality, whereas robust surveillance systems in urban areas improve the identification and reporting of such events (42, 52). In contrast, non-metropolitan populations may face increased vulnerability due to limited healthcare access and delayed emergency response, highlighting distinct but overlapping mechanisms of risk (10, 11). Research has indicated that the risk of heat-related mortality is around 3.3% greater in rural areas as compared to urban areas (53). Despite these indicators of increased vulnerability in rural settings, heat-related morbidity and mortality are likely underreported (54), largely due to limited healthcare access (55, 56), lower health insurance coverage (56, 57), and the absence of standardized criteria for classifying and documenting heat-related deaths (58, 59).

The finding that majority of deaths occurred at home underscores the rapid progression of heat-related cardiovascular events and suggests delays in recognition or access to care, emphasizing the need for early intervention and public awareness (12–14).

The recent increase in mortality seen in our analysis is consistent with prior research comparing effects of heat waves on cardiovascular mortality (5, 7, 31, 60, 61) and is likely reflective of broader climatic changes, including rising global temperatures and increasing frequency and severity of extreme heat events. Heat exposure contributes to cardiovascular mortality through multiple mechanisms, including dehydration, increased cardiac workload, electrolyte imbalance, and impaired thermoregulation (7, 31). These findings reinforce the growing intersection between climate change and cardiovascular health and highlight the need for targeted mitigation and adaptation strategies.

Public health interventions are essential to address this growing burden. Strategies such as heat warning systems, expansion of cooling centers, community outreach programs, and public education on early recognition of heat-related illness are critical, particularly for vulnerable populations (15–17). In addition, urban planning strategies, including increased green space and improved infrastructure, may reduce heat exposure at the population level (18, 19). Strengthening healthcare system preparedness during extreme heat events is also crucial to reduce preventable mortality.

Several limitations should be acknowledged. The use of International Classification of Diseases codes and death certificate may have led to misclassification or underreporting of cause of death. The CDC WONDER database does not include individual-level clinical and environmental data such as patient comorbid conditions, medication use, vital parameters, and socioeconomic status, all of which have a significant effect on mortality outcomes. Additionally, the ecological design precludes causal inference and introduces the potential for ecological fallacy. Changes in coding practices over time may also influence observed trends. Moreover, race-specific trends were restricted to the 2009–2024 period because race-related variables in earlier years were unreliable or inconsistently reported. Additionally, non-Hispanic American Indian or Alaska Native and non-Hispanic Asian or Pacific Islander populations were excluded from analyses due to unreliable or insufficient data reporting. Despite these limitations, the use of a large, nationally representative dataset and inclusion of multiple contributing causes enhances the robustness and relevance of our findings. To our knowledge, this is among the first national analyses to evaluate long-term trends in cardiovascular mortality incorporating heat exposure as a contributing cause using CDC WONDER data through 2024. These findings provide important insight into evolving epidemiological patterns and underscore the urgent need for targeted public health interventions in the context of a warming climate.

## 5 Conclusions

Heat-related cardiovascular mortality in the United States has increased substantially in recent years despite relatively stable long-term trends. Significant demographic and geographic disparities persist, particularly among males, Hispanic populations, and residents of the Western and Southern regions. These findings highlight the growing impact of climate-related heat exposure on cardiovascular health and underscore the need for targeted public health strategies, improved healthcare preparedness, and climate-adaptive interventions to mitigate preventable mortality.

## Data Availability

The original contributions presented in the study are included in the article/Supplementary Material, further inquiries can be directed to the corresponding author.
