# Supplementary figures and images for: Temporal trends and disparities in heat-related cardiovascular mortality in the United States (1999–2024): a CDC WONDER analysis
Source: Front Cardiovasc Med. 2026 Jun 24;13:1850364. doi: 10.3389/fcvm.2026.1850364 (PMC13341483; doi:10.3389/fcvm.2026.1850364)

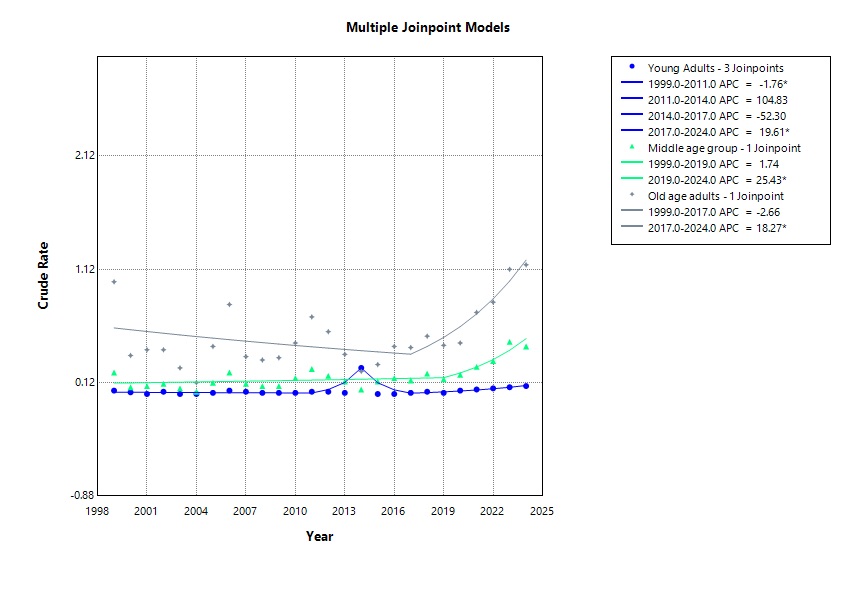

Supplement: Supplementary file 1 [file Image1.jpeg]

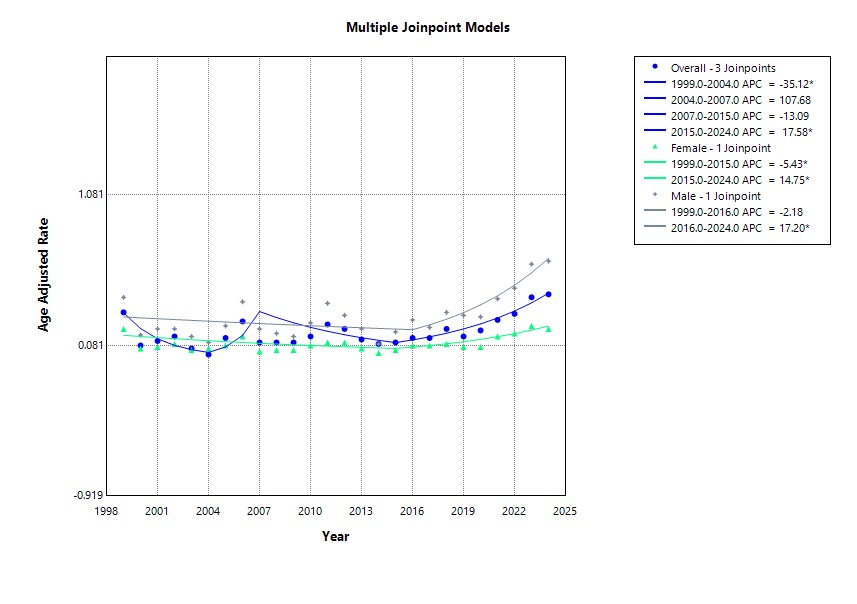

Supplement: Supplementary file 2 [file Image2.jpeg]

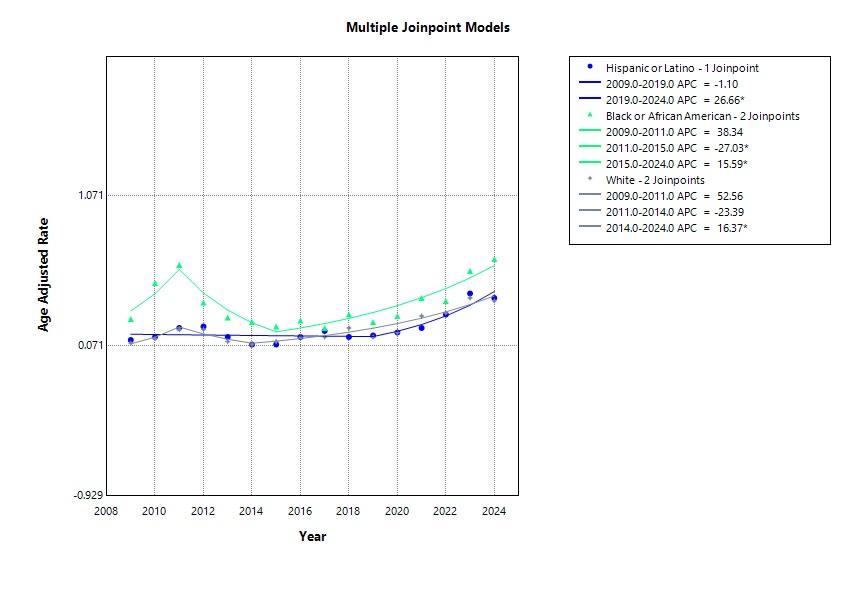

Supplement: Supplementary file 3 [file Image3.jpeg]

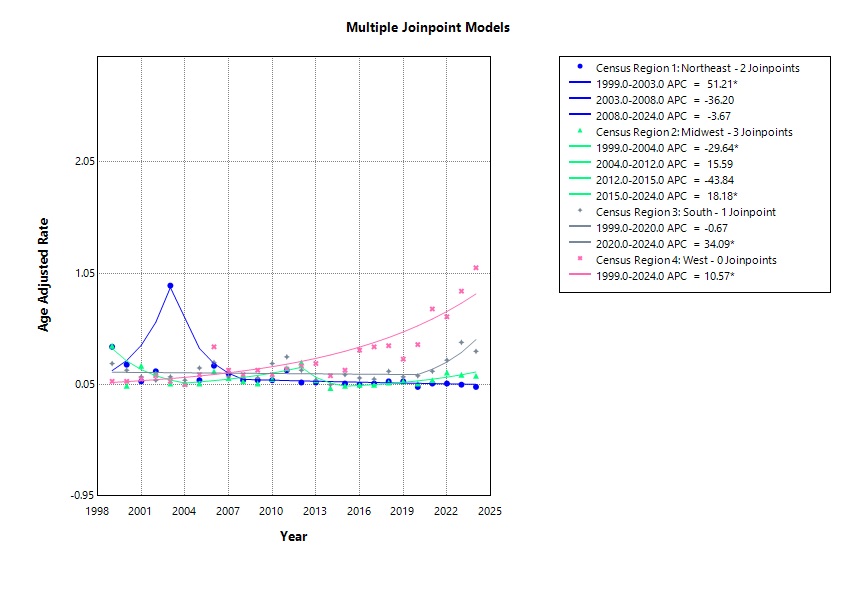

Supplement: Supplementary file 4 [file Image4.jpeg]

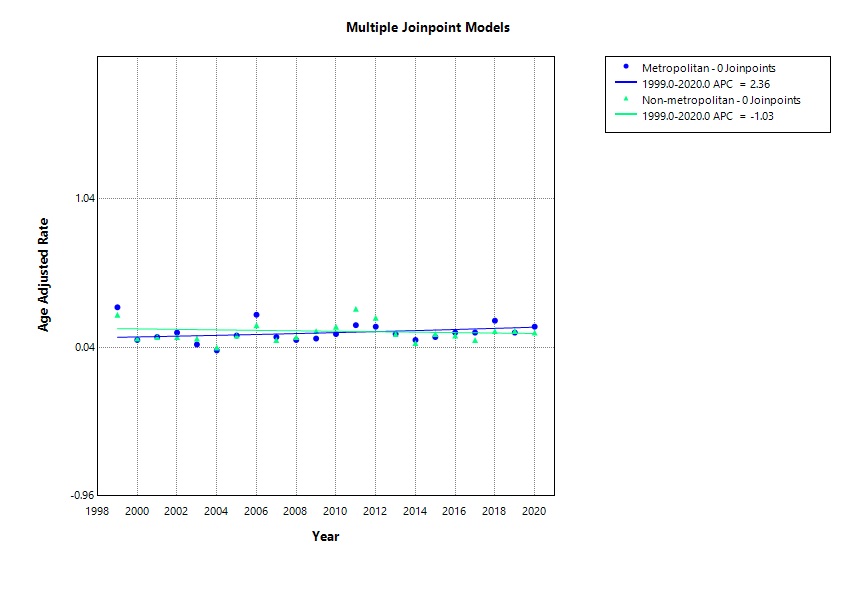

Supplement: Supplementary file 5 [file Image5.jpeg]
